# Effect of Active Lengthening and Shortening on Small-Angle X-ray Reflections in Skinned Skeletal Muscle Fibres

**DOI:** 10.3390/ijms22168526

**Published:** 2021-08-08

**Authors:** Venus Joumaa, Ian C. Smith, Atsuki Fukutani, Timothy R. Leonard, Weikang Ma, Srboljub M. Mijailovich, Thomas C. Irving, Walter Herzog

**Affiliations:** 1 Human Performance Laboratory, Faculty of Kinesiology, University of Calgary, Calgary, AB T2N1N4, Canada; vjoumaa@ucalgary.ca (V.J.); icsmith@ucalgary.ca (I.C.S.); leonard@ucalgary.ca (T.R.L.); 2 McCaig Institute for Bone and Joint Health, University of Calgary, Calgary, AB T2N1N4, Canada; 3 Faculty of Sport and Health Science, Ritsumeikan University, Kusatsu 525-8577, Japan; atsukifukutani@gmail.com; 4 The Biophysics Collaborative Access Team (BioCAT), Department of Biological Sciences, Illinois Institute of Technology, Chicago, IL 60616, USA; wma6@iit.edu (W.M.); irving@iit.edu (T.C.I.); 5 Department of Biological Sciences, Illinois Institute of Technology, Chicago, IL 60616, USA; smijailo@gmail.com

**Keywords:** equatorial X-ray reflections, meridional X-ray reflections, lattice spacing, shift in mass, force depression, residual force enhancement, stiffness

## Abstract

Our purpose was to use small-angle X-ray diffraction to investigate the structural changes within sarcomeres at steady-state isometric contraction following active lengthening and shortening, compared to purely isometric contractions performed at the same final lengths. We examined force, stiffness, and the 1,0 and 1,1 equatorial and M3 and M6 meridional reflections in skinned rabbit psoas bundles, at steady-state isometric contraction following active lengthening to a sarcomere length of 3.0 µm (15.4% initial bundle length at 7.7% bundle length/s), and active shortening to a sarcomere length of 2.6 µm (15.4% bundle length at 7.7% bundle length/s), and during purely isometric reference contractions at the corresponding sarcomere lengths. Compared to the reference contraction, the isometric contraction after active lengthening was associated with an increase in force (i.e., residual force enhancement) and M3 spacing, no change in stiffness and the intensity ratio I_1,1_/I_1,0_, and decreased lattice spacing and M3 intensity. Compared to the reference contraction, the isometric contraction after active shortening resulted in decreased force, stiffness, I_1,1_/I_1,0_, M3 and M6 spacings, and M3 intensity. This suggests that residual force enhancement is achieved without an increase in the proportion of attached cross-bridges, and that force depression is accompanied by a decrease in the proportion of attached cross-bridges. Furthermore, the steady-state isometric contraction following active lengthening and shortening is accompanied by an increase in cross-bridge dispersion and/or a change in the cross-bridge conformation compared to the reference contractions.

## 1. Introduction

When a skeletal muscle is actively lengthened or shortened, the resulting isometric steady-state force (after the transient effects of the length change are gone) is frequently greater or smaller, respectively, than the purely isometric contraction performed at the final muscle length without prior lengthening or shortening. These well-documented properties of skeletal muscle, termed residual force enhancement and force depression, respectively, have been observed consistently in muscle preparations ranging from single myofibrils to human muscles in vivo [1,2,3,4,5,6,7,8,9]. Residual force enhancement is known to increase with the magnitude of lengthening [1,10,11] and appears to be independent of the speed of lengthening [2]. Force depression increases with increasing shortening magnitudes [1,12,13] and decreases with increasing shortening speeds [1,12,13,14,15].

The underlying mechanisms of residual force enhancement and force depression are not understood. According to the cross-bridge theory [16], muscle contraction and active force production occur through the relative sliding of thin and thick filaments due to the cycling of actin–myosin cross-bridges. Residual force enhancement and force depression have been perplexing observations since the cross-bridge theory predicts that the steady-state isometric active force depends on the final muscle length and not on how the muscle reaches this final length [17]. The sarcomere length non-uniformity theory has been suggested to explain residual force enhancement and force depression [15]. This theory assumes that sarcomeres are not stable on the descending limb of the force length relationship [18], which results in sarcomeres being lengthened or shortened by different amounts during active lengthening or shortening [19]. This non-uniform behaviour leads to a situation in which sarcomeres, after attaining force equilibrium, produce tension that is greater or smaller than that produced at the corresponding length during an isometric contraction with sarcomeres of relatively uniform lengths. However, active lengthening and shortening experiments performed in single myofibrils, in which force and individual sarcomere lengths were measured [5,6,7,20,21], have demonstrated that residual force enhancement and force depression occur in the absence of increased sarcomere length non-uniformities and thus are likely intra-sarcomeric properties. This result is further supported by studies that showed residual force enhancement properties in single, mechanically isolated sarcomeres [22].

Within the framework of the cross-bridge theory, residual force enhancement and force depression could occur if active lengthening and shortening were able to induce nano-scale rearrangements of the muscle contractile filaments resulting in changes in the myofilament lattice spacing and the proportion and conformation of cross-bridges. Indirect attempts have been made to gain insight into the proportion of attached cross-bridges after active lengthening and shortening by measuring instantaneous stiffness in single muscle fibres [23,24,25]. However, although instantaneous stiffness, in some circumstances, may relate well to the proportion of attached cross-bridges, it cannot provide more than a rough glimpse at the structural changes in the sarcomeres and at the complex mechanism of residual force enhancement and force depression.

Despite an abundance of information regarding the properties of residual force enhancement and force depression, there has been no investigation of the effect of active lengthening and shortening on sarcomere structure, as reflected in small-angle X-ray diffraction patterns, at steady-state isometric contraction once transient forces have dissipated, compared to purely isometric contractions performed at the same final lengths. X-ray diffraction has provided a wealth of information about muscle ultrastructure [26,27,28,29,30,31] and is an ideal technique to provide structural information at the same time as physiological measurements in order to provide insight into the structural changes within the sarcomeres at steady-state isometric contraction after active lengthening and shortening.

The purpose of this study was to examine, using small-angle X-ray diffraction, the structural changes within the sarcomeres, at steady-state isometric contraction, after active lengthening and shortening compared to a purely isometric contraction performed at the same final length and thus gain insight into possible mechanisms for residual force enhancement and force depression.

We recorded X-ray diffraction patterns from skinned rabbit psoas muscle bundles both during purely isometric reference contractions performed at sarcomere lengths (SLs) of 3.0 µm and 2.6 µm, and at steady-state isometric contraction after active lengthening and shortening at SLs of 3.0 µm and 2.6 µm, respectively. We examined force, stiffness, and the 1,0 and 1,1 equatorial and M3 and M6 meridional X-ray reflections. Lattice spacing was calculated from the 1,0 equatorial reflections. In striated muscles, a shift in mass from the region of the thick filament to the region of the thin filament causes the intensity of the 1,1 reflection to increase and the intensity of the 1,0 reflection to decrease. The ratio of these intensities (I_1,1_/I_1,0_) may be used as a measure of the proximity of myosin cross-bridges to the thick or thin filaments [32,33]. The meridional reflection M3 is associated with the axial periodicity of the myosin heads, and if the cross-bridges undergo a structural change in their axial disposition or internal structure, this may be detected as a change in the intensity and spacing of the M3 reflection [30,34,35,36]. The spacing of the M6 reflection can be used to estimate thick filament backbone stiffness in response to either active or passive stress [28,36,37].

Compared to the reference contraction, active lengthening was associated with an increase in force (i.e., residual force enhancement) and M3 spacing, no change in stiffness and the intensity ratio I_1,1_/I_1,0_, and decreased lattice spacing and M3 intensity. Compared to the reference contraction, active shortening resulted in decreased force, stiffness, I_1,1_/I_1,0_, M3 and M6 spacings, and M3 intensity. These results suggest that residual force enhancement is achieved without an increase in the proportion of attached cross-bridges, and that force depression is accompanied by a decrease in the proportion of attached cross-bridges. Furthermore, active lengthening and shortening are accompanied by an increase in cross-bridge dispersion and/or a change in the cross-bridge conformation compared to the reference contractions.

## 2. Results

### 2.1. Active Lengthening Experiments

**Figure 1:**
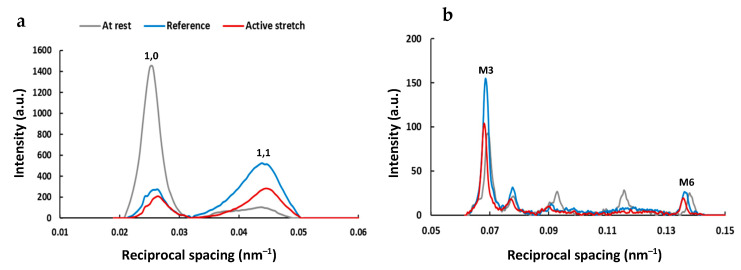
Typical 2D X-ray intensity peak profiles of the equatorial 1,0 and 1,1 and the meridional M3 and M6 reflections recorded at an average SL of 3.0 µm, at rest (gray) and at steady-state isometric contraction following a purely isometric reference contraction (blue) and an active lengthening contraction (red). (**a**) X-ray intensity peak profiles of the equatorial 1,0 and 1,1 reflections; (**b**) X-ray intensity peak profiles of the meridional M3 and M6 reflections. Intensity peak profiles were determined in 11 bundles for three conditions (rest, reference, and active stretch). Bundles underwent individual X-ray exposures of 0.5 s in total, and the X-ray patterns were collected as a continuous series of 18 ms frames separated by 2 ms in order to minimize radiation damage.

Active lengthening from an average SL of 2.6 µm to an average SL of 3.0 µm produced a residual force enhancement of 44 ± 10% (*p* = 0.009). There was no difference, however, in stiffness (*p* = 0.182) between the purely isometric and active lengthening states. Figure 1 shows typical 2D X-ray equatorial (Figure 1a) and meridional (Figure 1b) intensity peak profiles recorded at steady-state isometric contraction at an average SL of 3.0 µm after a purely isometric reference contraction and an active lengthening contraction.

**Figure 2:**
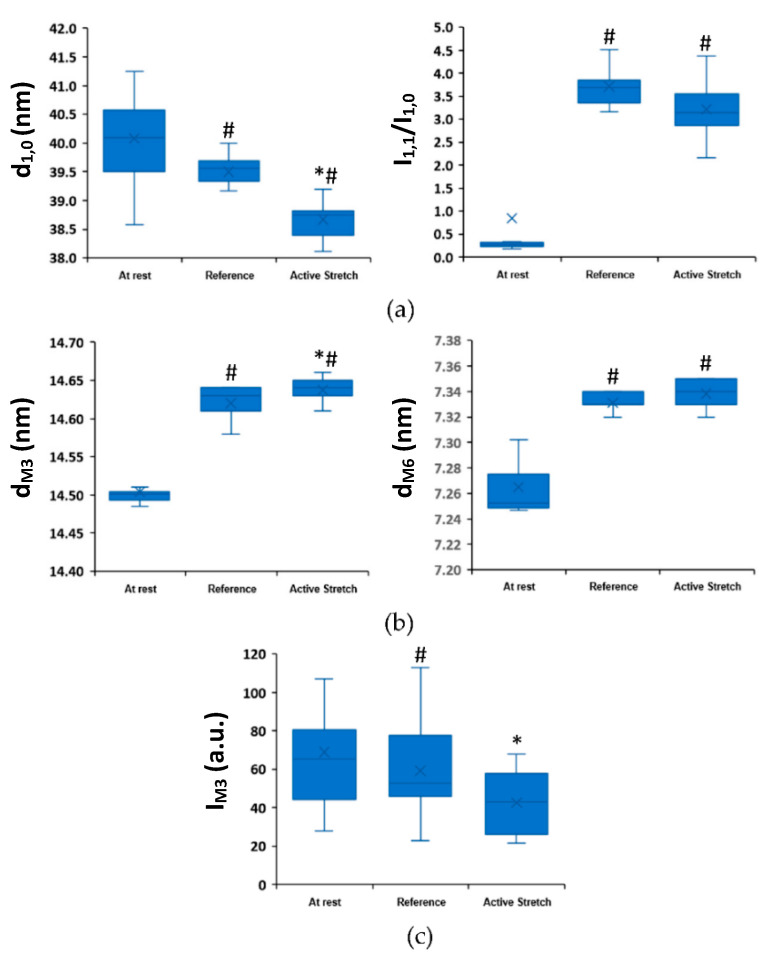
The equatorial and meridional reflections, at an average SL of 3.0 µm, at rest (Rest), and at steady-state isometric contraction following a purely isometric reference contraction (Reference) and an active lengthening contraction (Active Stretch). (**a**) Lattice spacing (d_1,0_) and the intensity ratio (I_1,1_/I_1,0_) measured from the equatorial reflections; (**b**) M3 and M6 spacings (d_M3_ and d_M6_) measured from the meridional reflections; (**c**) M3 intensity (I_M3_) measured from the meridional reflections. *: indicates a significant difference from the reference condition, #: indicates a significant difference from the rest condition. Data (*n* = 11 bundles) are shown as box plots, where whiskers show minimum and maximum values, boxes indicate the interquartile range from Q1 to Q3, and the median values are shown (x).

Active lengthening resulted in a reduction in lattice spacing (*p* = 0.015) but did not affect the equatorial intensity ratio, I_1,1_/I_1,0_ (*p =* 0.084), compared to the purely isometric contraction (Figure 2a). The spacing of the M3 meridional reflection significantly increased (*p* = 0.009) and the M3 peak intensity significantly decreased (*p* = 0.048) after active lengthening compared to the purely isometric contraction (Figure 2b,c).

### 2.2. Active Shortening Experiments

**Figure 3:**
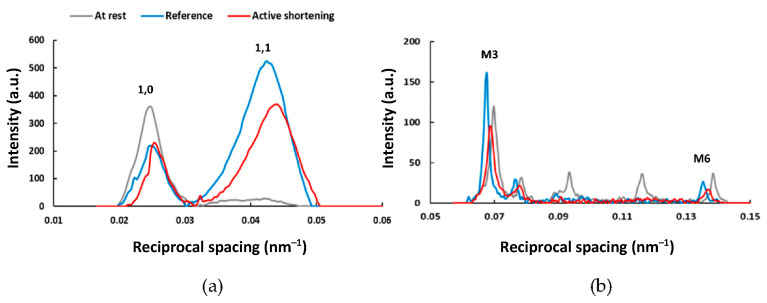
Typical 2D X-ray intensity peak profiles of the equatorial 1,0 and 1,1 and the meridional M3 and M6 reflections recorded at an average SL of 2.6 µm, at rest (gray), and at steady-state isometric contraction following a purely isometric reference contraction (blue) and an active shortening contraction (red). (**a**) X-ray intensity peak profiles of the equatorial 1,0 and 1,1 reflections; (**b**) X-ray intensity peak profiles of the meridional M3 and M6 reflections. Intensity peak profiles were determined in 10 bundles for three conditions (rest, reference, and active shortening). Bundles underwent individual X-ray exposures of 0.5 s in total, and the X-ray patterns were collected as a continuous series of 18 ms frames separated by 2 ms in order to minimize radiation damage.

Active shortening from an average SL of 3.0 µm to an average SL of 2.6 µm produced a force depression of 35 ± 8% (*p =* 0.008) and a stiffness depression of 40 ± 2% (*p = 0*.005). Figure 3 shows typical 2D X-ray equatorial (Figure 3a) and meridional (Figure 3b) intensity peak profiles recorded at the steady-state isometric contraction at an average SL of 2.6 µm after a purely isometric reference contraction and active shortening.

**Figure 4:**
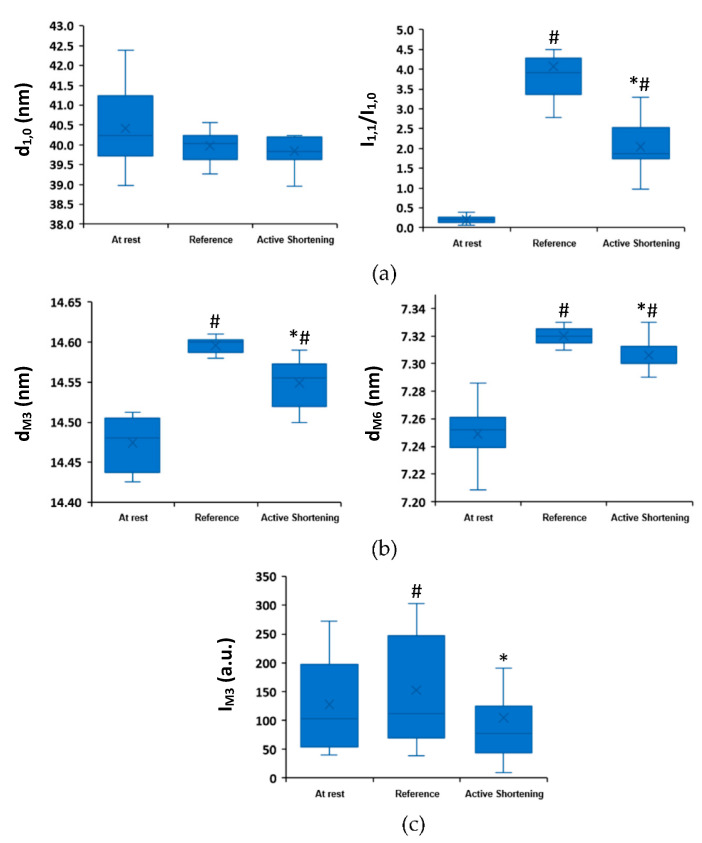
The equatorial and meridional reflections, at an average SL of 2.6 µm, at rest (Rest), and at steady-state isometric contraction following a purely isometric reference contraction (Reference) and an active shortening contraction (Active Shortening). (**a**) Lattice spacing (d_1,0_) and the intensity ratio (I_1,1_/I_1,0_) measured from the equatorial reflections; (**b**) M3 and M6 spacings (d_M3_ and d_M6_) measured from the meridional reflections; (**c**) M3 intensity (I_M3_) measured from the meridional reflections. *: indicates a significant difference from the reference condition, #: indicates a significant difference from the rest condition. Data (*n* = 10 bundles) are shown as box plots, where whiskers show minimum and maximum values, boxes indicate the interquartile range from Q1 to Q3, and the median values are shown (x).

Active shortening did not affect the lattice spacing (*p =* 0.99) but resulted in a reduction in I_1,1_/I_1,0_ (*p = 0*.036) compared to the purely isometric contraction (Figure 4a). The spacing of M3 and M6 and the peak intensity of M3 meridional reflections were significantly reduced (*p =* 0.021, 0.048, and 0.024, respectively) after active shortening compared to the purely isometric contraction (Figure 4b,c).

## 3. Discussion

The lengthening and shortening protocols used were effective in producing a substantial residual force enhancement (44 ± 10%) and force depression (35 ± 8%). The primary results of this study are that the residual force enhancement was achieved without an increase in the proportion of attached cross-bridges, and the force depression was accompanied by a decrease in the proportion of attached cross-bridges. These results are consistent between the mechanical (stiffness) measurements and the structural X-ray diffraction (I_1,1_/I_1,0_) measurements. Furthermore, the residual force enhancement and force depression appear to be accompanied by an increase in cross-bridge dispersion and/or a change in the conformation of the cross-bridges, as evidenced by a significant decrease in the intensity of the M3 meridional reflection [38].

Active lengthening induced a residual force enhancement, and active shortening resulted in force depression, as it has previously been shown [1,4,10,15,23,39,40]. The stiffness measurements performed in this study and by others [23,24,25] do not differ following the active lengthening and the purely isometric contractions and show a decrease in stiffness after active shortening, suggesting that while residual force enhancement is not accompanied by a change in the proportion of attached cross-bridges, force depression results in a decrease in the proportion of attached cross-bridges. Such inferences concerning the proportion of attached cross-bridges have been made in previous studies on force enhancement and depression, but stiffness measurements are associated with a series of non-trivial assumptions: for example, that the compliance of actin, myosin, titin, and other muscle structures [41,42] does not affect stiffness measurements or, at least, does not affect stiffness measurements differently between the contractile conditions tested here.

It has been shown using X-ray experiments that a shift in mass from the region of the thick filament to the region of the thin filament causes the intensity of the 1,1 equatorial reflection to increase and the intensity of the 1,0 equatorial reflection to decrease [33,43]. This mass shift has been generally interpreted as an increase in the formation of cross-bridges, that is, attachment of the myosin heads to the thin filaments [44,45,46], and has been shown to be closely correlated with the number of attached force-producing cross-bridges in permeabilized and intact contracting mammalian skeletal [43,47] and cardiac muscle [48,49]. More generally, the equatorial intensity ratio, I_1,1_/I_1,0_, can be used as a measure of the proximity of myosin heads to the thick or thin filaments [50,51,52]. We found that I_1,1_/I_1,0_ increased from the passive to the active states, indicating an increase in the proximity of myosin heads to the thin filament after activation. Furthermore, I_1,1_/I_1,0_ was not different between the steady-state isometric contraction following active lengthening and the purely isometric contraction and was reduced following active shortening compared to the purely isometric contractions, suggesting that there was no increase in the shift in mass from the thick to the thin filament after active lengthening, but a reduced shift in mass from the thick to the thin filament after active shortening compared to the corresponding purely isometric contractions. Similar results, i.e., an absence of an increase and a decrease in I_1,1_/I_1,0_ at steady-state isometric contraction after active lengthening and shortening, respectively, compared to the force before lengthening and shortening, have been shown previously by Amemiya et al. in 1988 [53]. Consequently, the combination of stiffness and X-ray diffraction measurements leads us to propose that the increase in force after active lengthening occurs without a significant change in the proportion of attached cross-bridges, while the decrease in force following active shortening is accompanied by a decrease in the proportion of attached cross-bridges.

The meridional reflections M3 and M6 provide precise measurements of the axial periodicity of the thick filament heads and backbone, respectively. An increase in the spacing of M3 and M6 upon activation has been documented for many years and has been explained as a force-induced lengthening of the myosin filament [28,30,54]. Active lengthening was associated with an increase in M3 spacing, while active shortening resulted in a decrease in M3 and M6 spacings, compared to the corresponding purely isometric contractions. This finding could be explained by the increase and decrease in force observed after active lengthening and shortening, respectively.

The intensity of M3 decreased at the isometric steady-state after active lengthening and shortening compared to the purely isometric contractions. Previous studies have suggested that the intensity of M3 comes primarily from the actin-attached myosin heads in the overlap region and that it has a strong dependence on the position of the lever arm of the actin-attached heads. Myosin heads in the non-overlap region are axially disordered and are thought to make only minor contributions to the intensity of the M3 reflection [28,35,54]. In active shortening experiments, where our data suggest a decrease in the proportion of actin-attached cross-bridges compared to the purely isometric contraction, a fall in the M3 intensity would be expected, as we observed. However, since active lengthening did not involve a change in the proportion of attached cross-bridges, the observed decrease in the M3 intensity cannot be explained solely by a reduction in the number of actin-attached myosin heads. An alternative explanation, that seems more likely, is that the dispersion of the actin-attached cross-bridges from their axial distribution significantly increased following active lengthening compared to the purely isometric contraction. Active lengthening could have caused the formation of several populations of cross-bridges of different conformations, with some having their lever arm tilted away from the perpendicular orientation characteristic of the M3 intensity [55]. It has been found previously that the intensity of M3 was reduced when active fibres were warmed from 4 to 24 °C, and this result was interpreted as an increase in the dispersion of cross-bridges during contractions at high temperatures [34]. It has been suggested [56,57] that the second head of the myosin dimer attaches to actin during active lengthening and thus leads to an increase in stiffness and force during stretch. The presence of a second cycling head, in addition to the initially attached one, at steady-state isometric contraction following active stretch, could potentially lead to changes in the configuration of the myosin heads and their lever arms and thus result in an increase in cross-bridge dispersion. However, this explanation is not consistent with our stiffness and intensity ratio results that suggest that steady-state isometric contraction following active lengthening is not accompanied by an increase in the proportion of attached cross-bridges. Furthermore, we believe that, although the attachment of the second head while the first head is still attached has been shown during active lengthening, the attachment of both heads during purely isometric contractions is highly unfavourable due to steric effects constraining the ability of the heads to cycle properly [58]. We also cannot exclude the possibility that the reduction in the M3 intensity after active shortening could be due to an increase in cross-bridge dispersion in addition to the observed reduction in the proportion of attached cross-bridges, as previous studies have reported an increase in cross-bridge dispersion following length perturbations imposed on fully activated fibres and muscles [38,53,59,60,61].

One of the mechanisms suggested to explain residual force enhancement and force depression is the development of sarcomere length non-uniformities following active lengthening and shortening [15]. The development of sarcomere length non-uniformities likely causes a large dispersion of cross-bridge forces because some cross-bridges would be in shortening conditions while others are in lengthening conditions. This is relevant during relaxation and muscle shortening where considerable sarcomere non-uniformity in the so-called chaotic phase of relaxation occurs [62]. In contrast, during lengthening, it is unlikely that stretch will result in significant sarcomere heterogeneity since stretching of cross-bridges likely causes an increase in the average cross-bridge force and a reduction in the detachment rates, and thus minor fluctuations in sarcomere forces along the muscle fibre. In addition, the increase in titin force expected as sarcomere lengths increase would be sufficient to stabilize these fluctuations. Nevertheless, since we determined the X-ray patterns at steady-state after shortening and lengthening, i.e., when sarcomere lengths are relatively stable (not during shortening and lengthening when the sarcomere length non-uniformities develop), there is no reason to believe that the presence of sarcomeres at different lengths necessarily leads to the cross-bridges being in various configurations (increased dispersion), and thus the increase in cross-bridge dispersion is not necessarily the result of an increase in sarcomere length non-uniformities at steady-state isometric contraction following active lengthening and shortening. Furthermore, other predictions of the sarcomere length non-uniformity theory were not confirmed in this study, such as the suggested decrease in stiffness after active lengthening [15]. Furthermore, experiments performed in single myofibrils where force and sarcomere lengths could be directly measured have shown that residual force enhancement and force depression occur without a significant increase in sarcomere length non-uniformities [5,6,20].

The results of this study provide support for the hypothesis proposed by Marechal and Plaghki, who suggested that active shortening might be associated with a stress-induced inhibition of cross-bridge formation in the newly formed overlap zone created during active shortening [13]. We have provided mechanical and structural evidence that when a muscle is actively shortened, the proportion of attached cross-bridges is reduced. According to Marechal and Plaghki (1979) [13], actin filaments entering the overlap region during shortening are strained by the tetanic stress, and therefore normal cross-bridge connections are compromised. An additional consideration suggested by multiscale modeling studies [63] is that during shortening, cross-bridges in the pre-power stroke state, A.M.D.Pi, will quickly transition to the post-power stroke state, A.M.D., completing the cross-bridge cycle, meaning that the net rate of cross-bridge detachment will be significantly increased during and following active shortening (negative strain), leading to a net reduction in the number of attached heads.

Another possible explanation for the reduction in the proportion of attached cross-bridges after active shortening could be a partial deactivation of the thin and thick filaments due to the reduction in titin-based passive tension, as titin becomes less strained during the shortening phase. Cazorla et al. [64] showed that the maximum active tension and calcium sensitivity are staringly influenced by titin-based passive tension. Titin-based passive tension at a given sarcomere length can be reduced by first stretching a relaxed muscle to a long length and then releasing it to the initial length. Tension in subsequent contractions is substantially reduced at low titin-based passive tensions [65]. This is consistent with the release protocol of our experiments, likely resulting in a reduction in passive force and a partial deactivation of the sarcomere, and therefore leading to a reduction in the number of bound cross-bridges and, consequently, force depression. Partial deactivation of the thick filament and/or the stress-induced inhibition of cross-bridges could result in the transition of the myosin heads into the super-relaxed state and thus reduce their availability for binding to actin. Additional small-angle X-ray experiments combined with the use of spatially explicit computational models that predict both physiological outputs and small-angle X-ray diffraction data are required to gain further insight into the mechanism(s) responsible for force depression.

The mechanism of residual force enhancement remains unclear. It has been suggested, for a long time, that residual force enhancement is likely caused by an active cross-bridge-based and a passive structural element-based mechanism [11,66]. The active component could be associated with the structural rearrangements observed in this study in the force enhanced compared to the isometric reference state, resulting in an increase in the average force per cross-bridge. One such structural rearrangement could be a change in the distribution of the cross-bridges between pre- and post-stroke states (i.e., the A.M.D.Pi and A.M.D states, respectively) during muscle lengthening. Multiscale modeling studies [63] suggest that during lengthening, the force and bond energy in actin-attached cross-bridges increase, particularly in the positively strained A.M.D.Pi cross-bridges. Furthermore, the number of A.M.D.Pi cross-bridges increases because the A.M.D heads are forced backwards through their cycle (reverse stroke). During lengthening, this large number of cross-bridges in the A.M.D.Pi state cannot complete the ATP-ase cycle and can detach only by cycling back from the A.M.D.Pi state to the M.D.Pi state, or forcefully detach due to the excess cross-bridge force relative to the actin–myosin bond strength. At the end of the lengthening, the observed tetanic force remains high because the A.M.D.Pi heads detach slowly and cannot proceed through the normal cross-bridge cycle since the stored energy from ATP hydrolysis could be less than the energy in the actin–myosin bonds if they were strained by power stroke and active lengthening [63]. This mechanism results in an increase in the force produced per cross-bridge without a change in the proportion of attached cross-bridges following active lengthening, and this is consistent with our stiffness and X-ray data, and with previous models suggesting structural rearrangements of the cross-bridges during and following active lengthening [67,68].

For the passive structural contribution to residual force enhancement, the filamentous sarcomeric protein titin has been implicated. It has been shown that calcium binds to titin upon activation and increases its stiffness when actively lengthened [69,70,71,72]. Furthermore, there is evidence that titin binds to actin [7,69,73,74,75], thereby shortening its I-band extensible region and increasing its stiffness. Additionally, it has been demonstrated that titin-based passive force induces stretch-based alterations in the structural arrangement and flexibility of the contractile filaments [76]. Therefore, we speculate that the increase in force after active lengthening could be, in part, the result of a titin-based increase in force when actively lengthened compared to the purely isometric contraction when titin is passively stretched before activation [7,77]. Future X-ray studies should focus on investigating the exact position of the cross-bridges relative to the myosin backbone and the conformation of titin before and after stretch, and on interpreting results within the framework of a muscle structural model that includes actin, myosin, and the elastic filament titin.

A limitation in this study is the use of thick and long muscle fibre bundles to perform the mechanical and X-ray experiments. The use of relatively large samples was required in order to obtain good X-ray diffraction patterns and reduce the radiation damage. However, this might have resulted in slow and inhomogeneous calcium diffusion along the length and thickness of the sample, and thus in heterogeneous sarcomere lengths between regions of the sample. Another limitation is the use of solutions that do not contain creatine phosphate and creatine kinase to replenish ATP. The accumulation of MgADP in the experimental chamber while the sample is contracting could impact the shortening velocity of cross-bridges [78]. However, we believe that the effect of heterogeneous calcium diffusion and the absence of an ATP backup system on our main results is minimal, since conditions were compared within the same bundles (i.e., active lengthening and shortening experiments were compared to their corresponding purely isometric contractions performed within the same bundle), and bundles were activated for relatively similar durations between conditions. Future research should attempt to address these limitations.

## 4. Materials and Methods

### 4.1. Skinned Muscle Bundle Preparation

Rabbits were euthanized by an intravenous injection of 1 mL of pentobarbital (240 mg/mL), a protocol approved by the University of Calgary’s Animal Care and Ethics Committee. Strips of psoas muscle were then dissected, tied to small wooden sticks, and stored in a skinning solution (see solutions below) for 12 h at 4 °C, then in a skinning–glycerol (50:50) solution at −20 °C for two weeks [6,7]. On the day of the experiments, skinned muscle bundles (mean ± SEM: length = 4.4 ± 0.3 mm and diameter = 388 ± 15 µm) were dissected from the skinned muscle strips and transferred to an experimental X-ray chamber with windows allowing exposure to X-rays and collection of the X-ray diffraction patterns. One end of the skinned bundle was glued to the hook of a length controller and the other end to the hook of a force transducer (Aurora Scientific Inc., Model 400A, Aurora, ON, Canada), allowing for control of muscle length and measurement of force, respectively. Sarcomere lengths were set at an average of 2.6 µm (initial bundle length, L_0_) using optical diffraction with a He-Ne laser. A rest period of 10 min was allowed between tests. All experiments were performed at room temperature (19–22 °C). Pilot experiments prior to the experiments reported here were performed in order to finalize the mechanical testing and X-ray radiation protocols and ensure that muscle bundles would not show signs of damage during the experiments.

### 4.2. Mechanical Testing Protocols

#### 4.2.1. Active Lengthening Experiments (*n* = 11)

- Passive lengthening from an average SL of 2.6 µm to an average SL of 3.0 µm

Fibre bundles were passively lengthened from an average SL of 2.6 µm to an average SL of 3.0 µm in relaxing solution in 2 s, held for 60 s, then exposed to X-rays for 0.5 s, and released to an average SL of 2.6 µm.

- Activation at an average SL of 2.6 µm and active lengthening to an average SL of 3.0 µm

**Figure 5:**
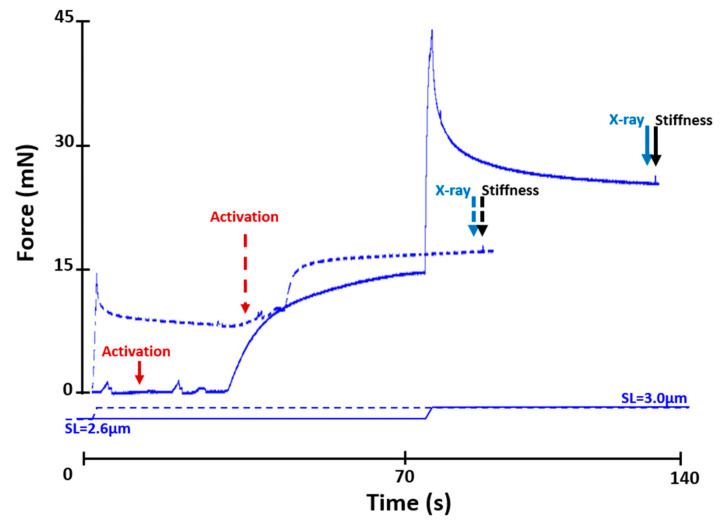
Force–time history of a reference (dashed line) and an active lengthening contraction (solid line). In the reference contraction, the fibre was passively lengthened from an average SL of 2.6 µm to an average SL of 3.0 µm and then activated. In the active lengthening contraction, the fibre was activated at an average SL of 2.6 µm and then actively lengthened to an average SL of 3.0 µm. Activation occurred when the relaxing solution in the testing chamber was replaced by a washing solution (free of EGTA and calcium) and then an activating solution (pCa 4.2). The noise in the graphs indicates the time when the solutions were changed. The dashed line arrows indicate events in the reference contraction, while the solid line arrows indicate events in the active lengthening contraction. The fibre was exposed to X-rays at steady-state isometric contraction following activation at a sarcomere length of 3.0 µm in the reference condition, and following active lengthening from an average SL of 2.6 to 3.0 µm in the active lengthening condition. Stiffness was measured after exposure to X-rays.

Fibre bundles were set at an average SL of 2.6 µm inside the mechanical chamber which was connected to a multi-valve syringe pump (Hamilton model 500) for remote solution changes. The muscle was activated at an average SL of 2.6 µm, then actively lengthened to an average SL of 3.0 µm in 2 s (at a speed of 7.7 % L_0_/s [15,25]), and held at this length for 60 s (Figure 5). Bundles were then exposed to X-rays for 0.5 s, deactivated, and released to an average SL of 2.6 µm.

- Activation at an average SL of 3.0 µm

Fibre bundles were passively stretched from an average SL of 2.6 µm to an average SL of 3.0 µm in 2 s, held until steady-state was reached, and then activated, exposed to X-rays for 0.5 s, deactivated, and released to an average SL of 2.6 µm (Figure 5).

Residual force enhancement was determined as the percent difference between the steady-state isometric force following active lengthening and the purely isometric reference force at an average SL of 3.0 µm.

#### 4.2.2. Active Shortening Experiments (*n* = 10)

- At rest at an average SL of 2.6 µm

Fibre bundles were set at an average SL of 2.6 µm in relaxing solution and exposed to X-rays for 0.5 s.

- Activation at an average SL of 3.0 µm and active shortening to an average SL of 2.6 µm

**Figure 6:**
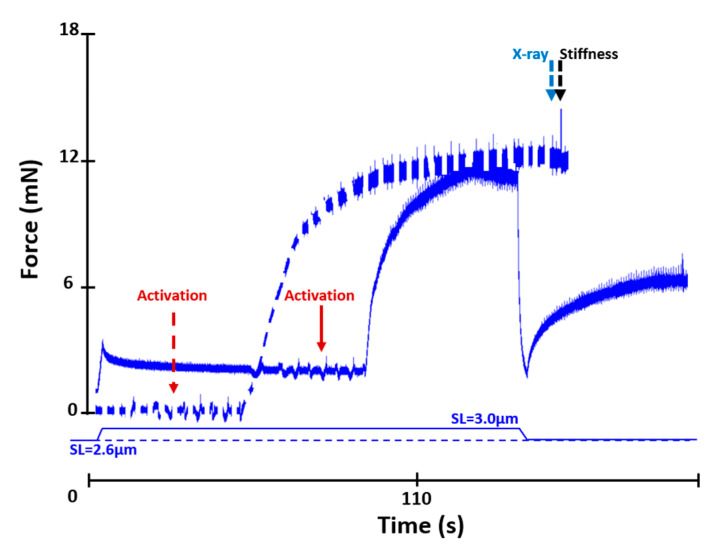
Force–time history of a reference (dashed line) and an active shortening contraction (solid line). In the reference contraction, the fibre was activated at an average SL of 2.6 µm. In the active shortening contraction, the fibre was passively stretched from an average SL of 2.6 µm to an average SL of 3.0 µm, activated, and then actively shortened to an average SL of 2.6 µm. Activation occurred when the relaxing solution in the testing chamber was replaced by a washing solution (free of EGTA and calcium) and then an activating solution (pCa 4.2). The noise in the graphs indicates the time when the solutions were changed. The dashed line arrows indicate events in the reference contraction, while the solid line arrows indicate events in the active shortening contraction. The fibre was exposed to X-rays at steady-state isometric contraction following activation at an average SL of 2.6 µm in the reference condition, and following active shortening from an average SL of 3.0 to 2.6 µm in the active shortening condition. Stiffness was measured after exposure to X-rays.

Fibre bundles were passively stretched from an average SL of 2.6 µm to an average SL of 3.0 µm in 2 s and held until steady-state was reached. Bundles were then activated and actively shortened to an average SL of 2.6 µm in 2 s (at a speed of 7.7 % L_0_/s [15,23,79]), held at this length for 60 s, and then exposed to X-rays for 0.5 s (Figure 6).

- Activation at an average SL of 2.6 µm

Fibre bundles were set at an average SL of 2.6 µm in relaxing solution and then activated and exposed to X-rays for 0.5 s at steady-state (Figure 6).

Force depression was determined as the percent difference between the steady-state isometric force following active shortening and the purely isometric reference force at an average SL of 2.6 µm.

#### 4.2.3. Stiffness Measurements

Fibre bundle stiffness was determined using a quick stretch–release protocol of 0.2% bundle length in 2 ms. Stiffness was measured once the isometric steady-state force had been reached after active lengthening and shortening, and during the purely isometric reference contractions (Figure 5 and Figure 6), always following X-ray exposure. Stiffness (instantaneous stiffness) was measured as the difference between the peak force reached after quick stretch and the force immediately before stretch, divided by the amplitude of stretch.

### 4.3. X-ray Data Collection and Analysis

X-ray diffraction experiments used the small angle instrument of the BioCAT beamline 18ID at the Advanced Photon Source, Argonne National Laboratory [80], using an apparatus similar to that described previously [76,81]. The X-ray beam energy was set to 12 keV (0.1033 nm wavelength) at an incident flux of ~10^13^ photons per second in the full beam. The X-ray beam was focused to ~130 × 30 µm at the detector. The specimen-to-detector distance was about 3 m. The experiment was controlled by an ASI 600A data acquisition and control system (Aurora Scientific Inc., Ontario, Canada) which was also used to collect force and muscle length data at a sampling rate of 10 kHz. During the exposure, the muscle samples were continuously oscillated in the beam at 10 mm/s to spread the X-ray dose along the sample length to minimize radiation damage. The X-ray patterns were collected as a continuous series of 18 ms frames separated by 2 ms in order to further minimize radiation damage, using a Pilatus 3 1 M pixel array detector (Dectris Inc., Baden-Daettwil, Switzerland). Images belonging to individual exposures (0.5 s total duration) were subsequently summed for further analysis.

#### Analysis of X-ray Diffraction Patterns

**Figure 7:**
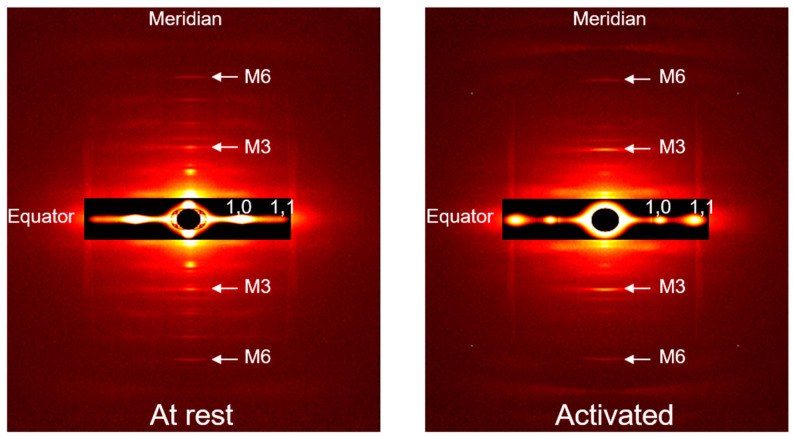
X-ray reflection patterns of a fibre bundle in the passive condition (at rest) and in the active condition (activated). Equatorial reflections 1,0 and 1,1 and meridional reflections M3 and M6 are indicated. Bundles were exposed to individual X-ray exposures of 0.5 s in total, and the X-ray patterns were collected as a continuous series of 18 ms frames separated by 2 ms in order to minimize radiation damage.

The data were analyzed using data analysis programs belonging to the MuscleX (Version 1.13) software package developed at BioCAT [82]. The images were quadrant folded and background subtracted using the “Quadrant fold” routine in MuscleX. The lattice spacing and intensity ratios (Figure 7) were measured using the “Equator” routine in MuscleX, as described previously [47]. The spacing and intensity of the M3 and M6 meridional reflections (Figure 7) were measured using the “Diffraction Centroids” routine in MuscleX, as described previously [36].

### 4.4. Statistical Analysis

Data were tested for normality using the Shapiro–Wilk test and were found to be normally distributed for some but not all parameters, and therefore we selected non-parametric tests to determine significant differences between groups. We used Wilcoxon matched-pair signed-rank to compare force and stiffness between the purely isometric and active lengthening and shortening states. We used the Friedman test to compare the X-ray parameters (lattice spacing, equatorial first-order intensity ratios, M3 and M6 spacings, and peak intensity of M3) between passive, reference, and active lengthening and shortening states. For significant (*p* < 0.05) results, a post hoc Wilcoxon matched-pair signed-rank with a Bonferroni correction was performed to determine the groups that differed. The acceptable level of significance was set at *p* < 0.05. Data are expressed as the mean ± SEM.

### 4.5. Solutions

Relaxing solution (in mM): potassium propionate (170), magnesium acetate (2.5), MOPS (20), K2EGTA (5), and ATP (2.5), pH 7.0. Washing solution (in mM): potassium propionate (185), magnesium acetate (2.5), MOPS (20), and ATP (2.5), pH 7.0. Activating solutions (in mM): potassium propionate (170), magnesium acetate (2.5), MOPS (10), ATP (2.5), and CaEGTA (5), pCa (–log [Ca2^+^]) of 4.2, pH 7.0 [25]. All solutions contained one tablet of protease inhibitors (Complete, Roche Diagnostics, QC, Canada) per 100 mL of solution.

## 5. Conclusions

We have provided structural and mechanical evidence that force depression is likely caused by a reduction in the proportion of attached cross-bridges following active shortening compared to a purely isometric contraction. We have also shown that residual force enhancement occurs in the absence of an increase in the proportion of attached cross-bridges compared to the purely isometric contraction. Furthermore, residual force enhancement and force depression are likely accompanied by an increase in cross-bridge dispersion and a change in their conformation. Additional X-ray research is required to gain further insight into the 3D structure of the contractile proteins following active lengthening and shortening

## Data Availability

Data will be made available upon request.
